# Protective Effects of Salivary Factors in Dental Caries in Diabetic Patients of Pakistan

**DOI:** 10.1155/2012/947304

**Published:** 2012-06-24

**Authors:** Muhammad Jawed, Rashid N. Khan, Syed M. Shahid, Abid Azhar

**Affiliations:** ^1^Department of Biochemistry, Liaquat College of Medicine and Dentistry, Karachi 75290, Pakistan; ^2^Department of Medicine, Liaquat College of Medicine and Dentistry, Karachi 75290, Pakistan; ^3^The Karachi Institute of Biotechnology & Genetic Engineering (KIBGE), University of Karachi, Karachi 75270, Pakistan

## Abstract

Salivary factors have been studied for their effects on the process of dental caries in patients of diabetes mellitus type 2. In this study, protective role of salivary pH, salivary flow rate, and salivary calcium is assessed in the patients of diabetes mellitus type 2 with dental caries. The samples of saliva were collected from 400 patients of diabetes mellitus type 2 and 300 age- and sex- matched controls after getting informed consent. All the subjects were classified into four groups according to age. The severity of dental caries was counted by decayed, missed, and filled teeth (DMFT) score. The salivary pH, flow rate, and calcium levels were found to be low in patients as compared to controls. The levels of fasting blood sugar, HbA1c, and DMFT score were found to be significantly high in patients than controls. The glycemic factors were significantly correlated with salivary factors indicating their influence on progression of caries in diabetes. On the basis of findings, it is concluded that the suitable salivary pH and flow rate may be regarded as main protective factors against dental caries in diabetes. Optimum level of salivary calcium may be responsible for continuous supply of calcium to arrest the demineralization and help reduce the occurrence of dental caries.

## 1. Introduction

Dental caries is one of the common disorders of human beings and is a serious public health issue in developing world. It usually occurs in children and adolescents and is the most common cause of tooth loss in younger population but can affect any age group [1, 2]. Dental caries has multiple causative factors [3, 4]. The hallmark of dental caries is demineralization which is initiated by acidogenic plaque flora and low salivary flow leading to slow clearance, poor buffering, and reduced supply of calcium to repair the altered dental tissues [5, 6]. An inverse relationship between rate of secretion of saliva and caries status has been reported [7, 8].

Dental caries has been more prevalent and even severe in diabetic patients than nondiabetics [9–12]. People with diabetes are more likely to develop periodontal infections, gum diseases, and tooth decay [13]. Saliva has been regarded as protective fluid against dental caries through its special properties and composition [14–16]. These include pH, flow rate, and calcium level [4, 17]. Approximately 5% of all patients seen in dental clinics are reported to have diabetes [18]. Epidemiological studies have supported the view that adequate level of calcium in saliva might inhibit dental carries by opposing the process of demineralization [19, 20]. Saliva can affect incidence of dental caries in many ways, primarily, as cleansing agent to minimize the accumulation of dental plaque, secondly, by reducing enamel solubility by continuous supply of minerals, particularly calcium, and finally, by buffering and antibacterial activity [21]. The constant rise in salivary calcium in low concentration is helpful in reducing the caries formation [22, 23]. The mechanism that regulates salivary calcium deposition is the pH. Significant decrease in local pH changes the chemical balance of the tooth surface and increases the solubility of hydroxyapatite [24]. Diabetes mellitus has been known to influence the salivary composition and function, eventually effecting oral cavity and dental health [25]. Common oral problems associated with diabetes mellitus include xerostomia, increased susceptibility to infections, salivary dysfunction, and dental caries [8, 26, 27]. Studies have shown that diabetic patients having tight glycemic control are found to have less decayed, missed, and filled tooth [28–30].

The current study has been designed to estimate and compare the salivary pH, salivary flow rate, and salivary calcium in diabetes mellitus type 2 patients with dental caries and in nondiabetic subjects with dental caries.

## 2. Subjects and Methods

The present study was carried out at the Department of Biochemistry, University of Karachi, in collaboration with Fatima Jinnah Dental College and Hospital, and Liaquat College of Medicine and Dentistry and Darul Sehat Hospital, Karachi. A total of 400 diabetes mellitus type 2 patients were selected from the OPDs of Fatima Jinnah Dental Hospital, Darul Sehat Hospital, and various other hospitals and clinics of Karachi, all of whom gave informed consent. The research project started after getting proper approval from the research and ethical committee of respective institutions. A total of 300 age- and sex-matched nondiabetic healthy individuals were selected as the control group from the general population.

Inclusion criteria include known diabetic type 2 patients of both sexes for at least 3 years and age between 26 and 65 years with a DMFT score of ≥5. All the subjects were free from any vascular complication of diabetes as well as systemic illness and were not taking any caries-preventive regimen like fluoride toothpaste, fluoride rinses, or NaF/calcium tablets. Subjects, who gave improper history about missed tooth or suffering from any type of xerostomia 3 or having any oral inflammatory problems, were excluded. Dental examination was done with the assistance of a dentist under natural light source. All subjects were divided into four groups (100 each) according to age as follows:

 group I: 30–35 years,
 group II: 36–40 years,
 group III: 41–45 years,
 group IV: ≥46 years.

About 8–10 mL of unstimulated mixed saliva was obtained from all subjects 2 hours after the breakfast and 10 min after mouthwash with deionized water several times. Total duration of saliva collection was also noted in minutes by stopwatch.

The saliva samples were centrifuged for 15 min at 3000 rpm. The clear supernatant of saliva was separated and stored at −20°C until analyzed. The salivary pH was measured electrometrically by digital pH meter HI 8014 (Hanna Instrument, USA). The calcium was estimated calorimetrically by using a kit (Ref. no. 995936) supplied by Quimica Clinica Aplicada SA (Amposta, Spain).

Results are presented as mean ± SEM. Statistical significance and difference from control and test values were evaluated by Student's *t*-test. The parametric one-way analysis of variance (ANOVA) was used to compare means of a quantitative variable between two or more groups when equal variances were assumed. *P* *values* of <0.001 were considered significant. Correlation coefficient and analysis were used to describe the effects of one variable on the other by Pearson's correlation test. All statistical analyses were done by using statistical package for social sciences (SPSS) version 14.0 for Windows (Chicago, IL, USA).

## 3. Results

The BMI, DBP, SBP, FBS, HbA1c, and DMFT levels were found to be significantly high (*P* < 0.001) in patients than controls (Table 1). The salivary pH, calcium, and flow rate were found to be significantly low (*P* < 0.001) as compared to controls (Table 2). All the parameters were statistically different in groups II, III, and IV (*P* < 0.001) except age. The comparison of means with respect to demographic features among the study groups of patients showed that the BMI, SBP, FBS, HbA1c, and DMFT values were significantly high (*P* < 0.001), whereas no significant difference was found in age and DBP (Table 3). The levels of salivary pH, calcium and flow rate were significantly low (*P* < 0.001) in patients of group II, III, and IV (Table 4).

**Figure 1:**
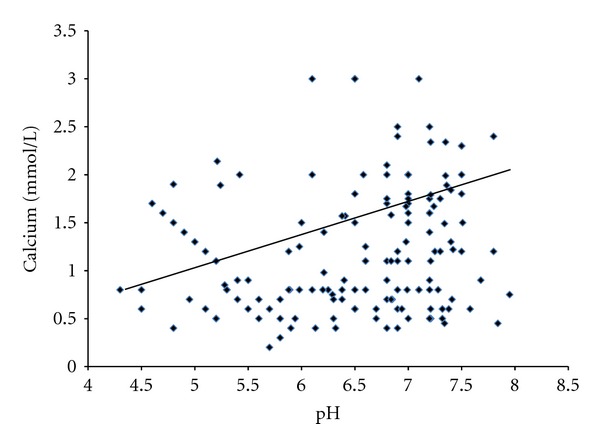
Correlation between salivary pH and calcium in patients (*r* = 0.64, *P* < 0.001).

**Figure 2:**
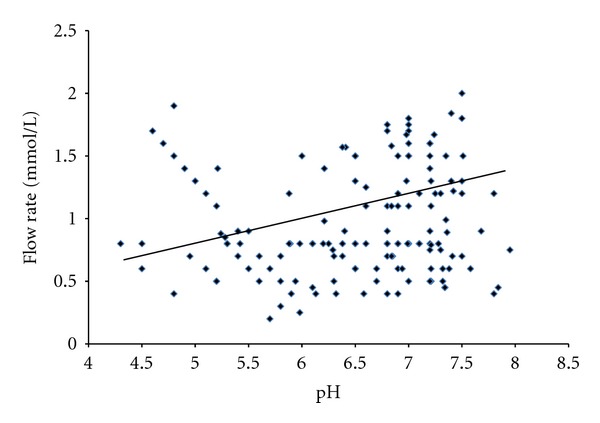
Correlation between salivary pH and flow rate in patients (*r* = 0.58, *P* < 0.001).

**Figure 3:**
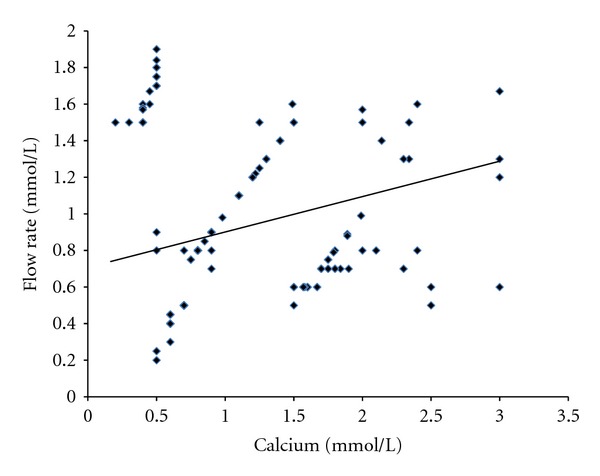
Correlation between salivary calcium and flow rate in patients (*r* = 0.71, *P* < 0.001).

The findings of this study also suggest that the salivary factors are associated and have a positive impact on each other. A strong positive and significant correlation was found out among the salivary pH, calcium, and flow rate indicating their impact and protective effects on them in the study groups of patients (Figures 1, 2, and 3).

## 4. Discussion

The protective role of salivary factors like pH, salivary flow rate, and salivary calcium has been evaluated since the middle of the last century [27, 29]. Most of the previous studies showed significantly reduced level of salivary pH in subjects of dental caries [15, 21]. Significantly lower values of salivary pH in dental caries patients were observed in this study, indicating favorable environment for the process of demineralization leading to cavity formation. It is suggested that the effect of low salivary pH is more in plaque which is close to the area of susceptible tooth surface [19, 30]. Calcium in saliva acts as chief mineral to prevent dissolution of teeth via its solubility constant and continuous supply to affected areas of teeth [30–32]. All the inorganic minerals present in serum are in continuous exchange phase with saliva around dental plaque and acting as reservoir of calcium to maintain adequate saturation level [20, 33].

Anticaries factors of saliva like optimum pH, rapid flow rate, and adequate level of calcium are severely affected by failure of blood glucose control by the patients suffering from diabetes mellitus type 2. These protective factors are seriously disturbed in all conditions of hyperglycemia [9, 25, 34]. Good control of blood sugar in diabetes has been found to be associated with low score of DMFT, reduced salivary flow rate, and pH [6]. The present study showed a significant difference (*P* < 0.001) of control subjects with DMFT score of 9.47 and patients with 12.01, which is comparable to earlier paper [10]. Similarly, the difference between the values of salivary flow rates in patients (1.06 mL/min) and in controls (2.38 mL/min) was significantly different (*P* < 0.001). The present study also indicates that the levels of salivary pH, flow rate, and calcium are significantly low in advanced group of diabetic patients. The process of demineralization has been checked by the suitable concentration of calcium, phosphate, and other inorganic ions around the affected teeth [19, 31]. The results explained the mechanism of remineralization on the basis of solubility of ionic products. The mineral composition of dental architecture would be more balanced, perfect, and resistant to be demineralized by the cariogenic potentials [35]. It has been estimated that the risk for dental caries is about 3-fold in patients with diabetes compared to nondiabetics [9, 36], which is also observed in the study presented. Despite the abundant evidence of more severe periodontal disease and dental caries, which may start at a younger age among diabetic patients than controls, the response to treatment seems to be equal in the diabetic and control groups. No differences in the short-term (from a couple of weeks to a few months) response to nonsurgical dental treatment were found between diabetic patients and controls [37, 38].

## 5. Conclusion

The process of demineralization is the main mechanism causing dental caries. There are many defensive roles of saliva against demineralization like optimum level of salivary pH, flow rate, and suitable concentration of calcium. The problem of dental caries is aggravated by deficiency of calcium particularly in diabetes mellitus type 2 patients.

Optimum concentration of calcium in saliva prevents dental caries and promotes remineralization, by giving strength and perfectness to the structure of teeth. Decreased salivary flow rate is associated with severity of dental caries. Optimum salivary flow rate is responsible for continuous removal of cariogenic factors from the oral environment. Results of the present study indicate that poor glycemic control and significantly increased value of HbA1c in diabetic patients are associated with more numbers of dental caries. Further investigation is required to evaluate the role of other parameters of saliva in subjects of diabetes with dental caries.

## Figures and Tables

**Table 1:** Demographic features in controls and patients.

Parameters	Controls (*n* = 300)	Patients (*n* = 400)
Age (years)	41.55 ± 11.25	40.94 ± 9.68
BMI (kg/m^2^)	24.38 ± 10.51	31.71 ± 6.25^∗^
DBP (mmHg)	79.65 ± 8.23	84.59 ± 9.24^∗^
SBP (mmHg)	124.33 ± 15.98	134.87 ± 13.68^∗^
FBS (mmol/L)	5.52 ± 2.61	13.25 ± 4.42^∗^
HbA1c (%)	4.88 ± 2.14	16.54 ± 3.20^∗^
DMFT	10.54 ± 3.38	14.25 ± 1.88^∗^

Values are mean ± SEM.

^∗^ *P* < 0.001 as compared to controls.

**Table 2:** Salivary factors in controls and patients.

Parameters	Controls (*n* = 300)	Patients (*n* = 400)
pH	7.18 ± 0.98	5.99 ± 0.24^∗^
Calcium (mmol/L)	1.49 ± 0.52	0.87 ± 0.06^∗^
Flow rate (mL/min)	2.58 ± 0.19	1.10 ± 0.02^∗^

Values are mean ± SEM.

*P < 0.001 as compared to controls.

**Table 3:** Comparison of demographic features within study groups by ANOVA.

Parameters	Group II (*P* value)	Group III (*P* value)	Group IV (*P* value)
Age (years)	0.268158	0.155025	0.322184
BMI (Kg/m^2^)	0.000024	0.000018	0.000039
DBP (mmHg)	0.000008	0.000174	0.000004
SBP (mmHg)	0.009120	0.000247	0.000019
FBS (mmol/L)	0.000037	0.000204	0.000017
HbA1c (%)	0.000155	0.000194	0.000510
DMFT	0.000112	0.000214	0.000038

**Table 4:** Comparison of salivary factors within study groups by ANOVA.

Parameters	Group II (*P* value)	Group III (*P* value)	Group IV (*P* value)
pH	0.000021	0.000104	0.000094
Calcium (mmol/L)	0.000024	0.000011	0.000006
Salivary flow rate (mL/min)	0.000018	0.001027	0.000054
